# Supplementary material for: Association between neutrophil count and the risk of cardiovascular disease: A community-based cohort study in Taiwan
Source: PLoS One. 2025 May 7;20(5):e0322645. doi: 10.1371/journal.pone.0322645 (PMC12057848; doi:10.1371/journal.pone.0322645)
Supplement: S1 Table — (DOCX) [file pone.0322645.s001.docx]

**S1 Table. Operational definition of covariate**

| **Covariates** | **Question in the questionnaire** | **Category** |
| --- | --- | --- |
| Sex |  | Women/Men |
| Age | (Years old) | 30-39, 40-49, 50-59, 60-69, >70 |
| Current smoking status | Do you have smoking habit? 0. No; 1. Yes; 2. Previously yes, currently no  1=current smoker  0, 2 = non-current smoker | Current/Non-current smoker |
| Current drinking status | Do you have drinking habit? 0. No; 1. Yes; 2. Previously yes, currently no  1=current drinking  0, 2 = non-current drinking | Current/Non-current drinking |
|  | **unit** |  |
| Fasting plasma glucose | (mg/dL) | Continuous variable |
| Total cholesterol | (mg/dL) | Continuous variable |
| Triglycerides | (mg/dL) | Continuous variable |
| High-density lipoprotein cholesterol | (mg/dL) | Continuous variable |
| Low-density lipoprotein cholesterol | (mg/dL) | Continuous variable |
| Systolic blood pressure | (mmHg) | Continuous variable |
| Body Mass Index |  | Continuous variable |
