# Supplementary material for: Association between neutrophil count and the risk of cardiovascular disease: A community-based cohort study in Taiwan
Source: PLoS One. 2025 May 7;20(5):e0322645. doi: 10.1371/journal.pone.0322645 (PMC12057848; doi:10.1371/journal.pone.0322645)
Supplement: S2 Table — (DOCX) [file pone.0322645.s002.docx]

**S2 Table. Baseline characteristics of participants by red blood cell**

|  | **Total** | **Red blood cell** | | | |  |
| --- | --- | --- | --- | --- | --- | --- |
| **Characteristics** |  | **Q1** | **Q2** | **Q3** | **Q4** | ***p* value** |
|  |  | **0.86-4.28**  **(x10^3^/uL)** | **4.28-4.62**  **(x10^3^/uL)** | **4.62-5.03**  **(x10^3^/uL)** | **5.03-9.60**  **(x10^3^/uL)** |  |
|  | **n(%)** | **n(%)** | **n(%)** | **n(%)** | **n(%)** |  |
| **Age** |  |  |  |  |  | <0.001 |
| 35–64 years old | 2,318 (78.4) | 514 (69.8) | 568 (77.9) | 606 (80.9) | 630 (85.0) |  |
| ≥65 years old | 637 (21.6) | 222 (30.2) | 161 (22.1) | 143 (19.1) | 111 (15.0) |  |
| **Sex** |  |  |  |  |  |  |
| Woman | 1,581 (53.5) | 577 (78.4) | 475 (65.2) | 313 (41.8) | 216 (29.2) | <0.001 |
| Current smoker | 913 (30.9) | 127 (17.3) | 181 (24.8) | 289 (38.6) | 316 (42.7) | <0.001 |
| Alcohol use | 703 (23.8) | 102 (13.9) | 136 (18.7) | 207 (27.6) | 258 (34.8) | <.0001 |
|  | **mean**±**SD** | **mean**±**SD** | **mean**±**SD** | **mean**±**SD** | **mean**±**SD** |  |
| Body mass index (kg/m^2^) | 23.5±3.4 | 22.7±3.3 | 23.4±3.4 | 23.6±3.4 | 24.2±3.4 | <.0001 |
| Systolic blood pressure (mmHg) | 125±20.2 | 123.7±20.4 | 125.9±21.6 | 125±19.9 | 125.2±18.6 | 0.19 |
| Diastolic blood pressure (mmHg) | 77±11.1 | 74.7±10.4 | 77±11.4 | 77.8±11.2 | 78.6±10.9 | <0.001 |
| Fasting plasma glucose (mg/dL) | 109.8±31.3 | 104.6±21.3 | 108.1±28 | 112.2±35.3 | 114.1±37.2 | <0.001 |
| Total cholesterol (mg/dL) | 196.8±44.6 | 197±45.3 | 195±43.3 | 197.3±45.2 | 197.9±44.7 | 0.63 |
| Triglycerides (mg/dL) | 125.1±94.9 | 105.8±82.7 | 118.7±89.4 | 134.9±101.4 | 140.5±100.7 | <0.001 |
| High-density lipoprotein cholesterol (mg/dL) | 47.6±12.4 | 50.6±12.7 | 48.3±12.7 | 46.1±11.9 | 45.7±11.9 | <0.001 |
| Low-density lipoprotein cholesterol (mg/dL) | 137±43.5 | 134.2±43.6 | 135.1±42.2 | 139.1±44.3 | 139.4±43.7 | 0.036 |

**Abbreviations:** SD, standard deviation
