# Supplementary material for: Association between neutrophil count and the risk of cardiovascular disease: A community-based cohort study in Taiwan
Source: PLoS One. 2025 May 7;20(5):e0322645. doi: 10.1371/journal.pone.0322645 (PMC12057848; doi:10.1371/journal.pone.0322645)
Supplement: S3 Table — (DOCX) [file pone.0322645.s003.docx]

**S3 Table. Baseline characteristics of participants by white blood cell**

|  | **Total** | **White blood cell** | | | | |  | |
| --- | --- | --- | --- | --- | --- | --- | --- | --- |
| **Characteristics** |  | **Q1** | **Q2** | **Q3** | **Q4** | ***p* value** | |  |
|  |  | **2.1-5.1**  **(x10^3^/uL)** | **5.1-6.1**  **(x10^3^/uL)** | **6.1-7.2**  **(x10^3^/uL)** | **7.2-17.6**  **(x10^3^/uL)** |  |  |  |
|  | **n(%)** | **n(%)** | **n(%)** | **n(%)** | **n(%)** |  | |  |
| **Age** |  |  |  |  |  | 0.13 | |  |
| 35–64 years old | 2,318 (78.4) | 534 (77.5) | 594 (76.2) | 583 (80.9) | 607 (79.4) |  | |  |
| ≥65 years old | 637 (21.6) | 155 (22.5) | 186 (23.9) | 138 (19.1) | 158 (20.7) |  | |  |
| **Sex** |  |  |  |  |  |  | |  |
| Woman | 1,581 (53.5) | 434 (63.0) | 431 (55.3) | 373 (51.7) | 343 (44.8) | <0.001 | |  |
| Current smoker | 913 (30.9) | 166 (24.1) | 209 (26.8) | 228 (31.6) | 310 (40.5) | <0.001 | |  |
| Alcohol use | 703 (23.8) | 140 (20.3) | 174 (22.3) | 181 (25.1) | 208 (27.2) | 0.011 | |  |
|  | **mean**±**SD** | **mean**±**SD** | **mean**±**SD** | **mean**±**SD** | **mean**±**SD** |  | |  |
| Body mass index (kg/m^2^) | 23.5±3.4 | 22.6±3 | 23.3±3.4 | 23.8±3.4 | 24.1±3.6 | <0.001 | |  |
| Systolic blood pressure (mmHg) | 125±20.2 | 123.4±19.9 | 123.9±20 | 126.3±20.6 | 126.2±20.1 | 0.006 | |  |
| Diastolic blood pressure (mmHg) | 77±11.1 | 75.7±10.9 | 76.4±11 | 77.7±11.3 | 78.2±10.8 | <0.001 | |  |
| Fasting plasma glucose (mg/dL) | 109.8±31.3 | 106.3±26.4 | 108.5±30 | 110.7±33 | 113.2±34.6 | <0.001 | |  |
| Total cholesterol (mg/dL) | 196.8±44.6 | 191±44.9 | 195.3±44.3 | 197.3±42.6 | 203.1±45.9 | <0.001 | |  |
| Triglycerides (mg/dL) | 125.1±94.9 | 99.7±69.3 | 117.3±85.6 | 132.6±103.3 | 148.7±108.2 | <0.001 | |  |
| High-density lipoprotein cholesterol (mg/dL) | 47.6±12.4 | 50.2±12.9 | 48.3±12 | 46.5±12.5 | 45.8±12 | <0.001 | |  |
| Low-density lipoprotein cholesterol (mg/dL) | 137±43.5 | 129.1±43.3 | 135.1±43 | 138.4±42.1 | 144.7±44.3 | <0.001 | |  |

**Abbreviations:** SD, standard deviation
