# Supplementary material for: Association between neutrophil count and the risk of cardiovascular disease: A community-based cohort study in Taiwan
Source: PLoS One. 2025 May 7;20(5):e0322645. doi: 10.1371/journal.pone.0322645 (PMC12057848; doi:10.1371/journal.pone.0322645)
Supplement: S4 Table — (DOCX) [file pone.0322645.s004.docx]

**S4 Table. Baseline characteristics of participants by lymphocyte count**

|  | **Total** | **Lymphocyte count** | | | | |  | |
| --- | --- | --- | --- | --- | --- | --- | --- | --- |
| **Characteristics** |  | **Q1** | **Q2** | **Q3** | **Q4** | ***p* value** | |  |
|  |  | **0.4-1.8**  **(x10^3^/uL)** | **1.8-2.2**  **(x10^3^/uL)** | **2.2-2.6**  **(x10^3^/uL)** | **2.6-71.7**  **(x10^3^/uL)** |  |  |  |
|  | **n(%)** | **n(%)** | **n(%)** | **n(%)** | **n(%)** |  | |  |
| **Age** |  |  |  |  |  | <0.001 | |  |
| 35–64 years old | 2,318 (78.4) | 539 (73.3) | 548 (76.9) | 513 (80.3) | 718 (82.7) |  | |  |
| ≥65 years old | 637 (21.6) | 196 (26.7) | 165 (23.1) | 126 (19.7) | 150 (17.3) |  | |  |
| **Sex** |  |  |  |  |  |  | |  |
| Woman | 1,581 (53.5) | 411 (55.9) | 402 (56.4) | 325 (50.9) | 443 (51) | 0.046 | |  |
| Current smoker | 913 (30.9) | 216 (29.4) | 181 (25.4) | 211 (33) | 305 (35.1) | <0.001 | |  |
| Alcohol use | 703 (23.8) | 154 (21) | 161 (22.6) | 159 (24.9) | 229 (26.4) | 0.06 | |  |
|  | **mean**±**SD** | **mean**±**SD** | **mean**±**SD** | **mean**±**SD** | **mean**±**SD** |  | |  |
| Body mass index (kg/m^2^) | 23.5±3.4 | 22.7±3.2 | 23.2±3.2 | 23.8±3.6 | 24.2±3.5 | <0.001 | |  |
| Systolic blood pressure (mmHg) | 125±20.2 | 124.5±20.7 | 124±20.1 | 125.1±20.5 | 126.1±19.5 | 0.18 | |  |
| Diastolic blood pressure (mmHg) | 77±11.1 | 75.8±11.4 | 76.3±11.1 | 77.4±10.8 | 78.4±10.8 | <0.001 | |  |
| Fasting plasma glucose (mg/dL) | 109.8±31.3 | 107.6±29.5 | 107.9±29.1 | 109.3±33.3 | 113.5±32.8 | <0.001 | |  |
| Total cholesterol (mg/dL) | 196.8±44.6 | 191.5±43.7 | 194.3±42.8 | 199±48.2 | 201.8±43.6 | <0.001 | |  |
| Triglycerides (mg/dL) | 125.1±94.9 | 104.9±76.2 | 110.7±71.9 | 131.5±104.3 | 149.2±111.2 | <0.001 | |  |
| High-density lipoprotein cholesterol (mg/dL) | 47.6±12.4 | 49.7±13.1 | 49.3±12.4 | 46.2±12.1 | 45.6±11.7 | <0.001 | |  |
| Low-density lipoprotein cholesterol (mg/dL) | 137±43.5 | 129.8±42.5 | 133.4±41.8 | 140.6±45.8 | 143.3±42.9 | <0.001 | |  |

**Abbreviations:** SD, standard deviation
