# Supplementary material for: Association between neutrophil count and the risk of cardiovascular disease: A community-based cohort study in Taiwan
Source: PLoS One. 2025 May 7;20(5):e0322645. doi: 10.1371/journal.pone.0322645 (PMC12057848; doi:10.1371/journal.pone.0322645)
Supplement: S5 Table — (DOCX) [file pone.0322645.s005.docx]

**S5 Table. Baseline characteristics of participants by platelet**

|  | **Total** | **Platelet** | | | | |  | |
| --- | --- | --- | --- | --- | --- | --- | --- | --- |
| **Characteristics** |  | **Q1** | **Q2** | **Q3** | **Q4** | ***p* value** | |  |
|  |  | **1.6-21.1**  **(x10^3^/uL)** | **21.1-25.8**  **(x10^3^/uL)** | **25.8-30.9**  **(x10^3^/uL)** | **30.9-109.5**  **(x10^3^/uL)** |  |  |  |
|  | **n(%)** | **n(%)** | **n(%)** | **n(%)** | **n(%)** |  | |  |
| **Age** |  |  |  |  |  | <0.001 | |  |
| 35–64 years old | 2,318 (78.4) | 537 (73.8) | 580 (77.4) | 585 (79.9) | 616 (82.6) |  | |  |
| ≥65 years old | 637 (21.6) | 191 (26.2) | 169 (22.6) | 147 (20.1) | 130 (17.4) |  | |  |
| **Sex** |  |  |  |  |  |  | |  |
| Woman | 1,581 (53.5) | 345 (47.4) | 369 (49.3) | 417 (57) | 450 (60.3) | <0.001 | |  |
| Current smoker | 913 (30.9) | 263 (36.1) | 251 (33.5) | 218 (29.8) | 181 (24.3) | <0.001 | |  |
| Alcohol use | 703 (23.8) | 177 (24.3) | 187 (25) | 169 (23.1) | 170 (22.8) | 0.73 | |  |
|  | **mean**±**SD** | **mean**±**SD** | **mean**±**SD** | **mean**±**SD** | **mean**±**SD** |  | |  |
| Body mass index (kg/m^2^) | 23.5±3.4 | 23.2±3.4 | 23.4±3.5 | 23.4±3.4 | 23.8±3.4 | 0.005 | |  |
| Systolic blood pressure (mmHg) | 125±20.2 | 124.9±21.1 | 125.7±20.7 | 124.7±19.7 | 124.7±19.1 | 0.74 | |  |
| Diastolic blood pressure (mmHg) | 77±11.1 | 76.8±11.4 | 76.7±11.1 | 77±11.1 | 77.6±10.7 | 0.44 | |  |
| Fasting plasma glucose (mg/dL) | 109.8±31.3 | 109.6±35.6 | 107.8±27.3 | 109.2±27.9 | 112.4±33.7 | 0.039 | |  |
| Total cholesterol (mg/dL) | 196.8±44.6 | 184.9±44.7 | 192.5±41.5 | 200.2±43.2 | 209.5±45.4 | <0.001 | |  |
| Triglycerides (mg/dL) | 125.1±94.9 | 114.6±88.3 | 118.7±83.6 | 129.8±103 | 137.1±101.8 | <0.001 | |  |
| High-density lipoprotein cholesterol (mg/dL) | 47.6±12.4 | 47.9±13 | 47.4±12.1 | 47.4±12.2 | 47.9±12.4 | 0.75 | |  |
| Low-density lipoprotein cholesterol (mg/dL) | 137±43.5 | 125±43.3 | 133.1±41 | 140.6±41.9 | 149±44.2 | <0.001 | |  |

**Abbreviations:** SD, standard deviation
