# Supplementary material for: Association between neutrophil count and the risk of cardiovascular disease: A community-based cohort study in Taiwan
Source: PLoS One. 2025 May 7;20(5):e0322645. doi: 10.1371/journal.pone.0322645 (PMC12057848; doi:10.1371/journal.pone.0322645)
Supplement: S6 Table — (DOCX) [file pone.0322645.s006.docx]

**S6 Table. The cardiovascular disease incidence according to the quartiles of red blood cell**

| **Variables** | **Q1** | **Q2** | **Q3** | **Q4** |  |
| --- | --- | --- | --- | --- | --- |
| Participants | 736 | 729 | 749 | 741 |  |
| Person-years | 12,691 | 13,040 | 13,174 | 13,420 |  |
| Events | 91 | 94 | 117 | 98 |  |
| Incidence rate | 7.17 | 7.21 | 8.88 | 7.30 |  |
| **Hazard ratio (95% CI)** | | | | | ***p* for trend** |
| Model 1 | Ref. | 1.13  (0.84-1.51) | 1.36  (1.02-1.81) | 1.31  (0.96-1.78) | 0.06 |
| Model 2 | Ref. | 1.03  (0.77-1.39) | 1.21  (0.91-1.63) | 1.13  (0.82-1.54) | 0.36 |
| Model 3 | Ref. | 0.94  (0.70-1.26) | 1.08  (0.80-1.45) | 0.95  (0.69-1.31) | 0.90 |

model 1: adjusted for age and sex; model 2: adjusted for model 1, body mass index, current smoker, alcohol use; model 3: adjusted for model 2, systolic blood pressure, fasting plasma glucose, total cholesterol, high density lipoprotein; low density lipoprotein

**Abbreviations:** CI, confidence interval
