# Supplementary material for: Association between neutrophil count and the risk of cardiovascular disease: A community-based cohort study in Taiwan
Source: PLoS One. 2025 May 7;20(5):e0322645. doi: 10.1371/journal.pone.0322645 (PMC12057848; doi:10.1371/journal.pone.0322645)
Supplement: S7 Table — (DOCX) [file pone.0322645.s007.docx]

**S7 Table. The cardiovascular disease incidence according to the quartiles of white blood cell**

| **Variables** | **Q1** | **Q2** | **Q3** | **Q4** |  |
| --- | --- | --- | --- | --- | --- |
| Participants | 689 | 780 | 721 | 765 |  |
| Person-years | 12,461 | 13,902 | 12,814 | 13,147 |  |
| Events | 73 | 101 | 105 | 121 |  |
| Incidence rate per 1000-person years | 5.86 | 7.27 | 8.19 | 9.20 |  |
| **Hazard ratio (95% CI)** | | | | | ***p* for trend** |
| Model 1 | Ref. | 1.28  (0.95-1.73) | 1.50  (1.11-2.02) | 1.75  (1.31-2.34) | <0.001 |
| Model 2 | Ref. | 1.21  (0.89-1.64) | 1.37  (1.01-1.85) | 1.57  (1.17-2.11) | 0.002 |
| Model 3 | Ref. | 1.14  (0.84-1.54) | 1.20  (0.88-1.62) | 1.35  (1.00-1.83) | 0.043 |

model 1: adjusted for age and sex; model 2: adjusted for model 1, body mass index, current smoker, alcohol use; model 3: adjusted for model 2, systolic blood pressure, fasting plasma glucose, total cholesterol, high density lipoprotein; low density lipoprotein

**Abbreviations:** CI, confidence interval
