# Supplementary material for: Association between neutrophil count and the risk of cardiovascular disease: A community-based cohort study in Taiwan
Source: PLoS One. 2025 May 7;20(5):e0322645. doi: 10.1371/journal.pone.0322645 (PMC12057848; doi:10.1371/journal.pone.0322645)
Supplement: S8 Table — (DOCX) [file pone.0322645.s008.docx]

**S8 Table. The cardiovascular disease incidence according to the quartiles of lymphocyte count**

| **Variables** | **Q1** | **Q2** | **Q3** | **Q4** |  |
| --- | --- | --- | --- | --- | --- |
| Participants | 735 | 713 | 639 | 868 |  |
| Person-years | 12,366 | 12,605 | 11,550 | 15,804 |  |
| Events | 78 | 103 | 92 | 127 |  |
| Incidence rate per 1000-person years | 6.31 | 8.17 | 7.97 | 8.04 |  |
| **Hazard ratio (95% CI)** | | | | | ***p* for trend** |
| Model 1 | Ref. | 1.36  (1.01-1.82) | 1.44  (1.07-1.95) | 1.56  (1.17-2.07) | 0.003 |
| Model 2 | Ref. | 1.30  (0.97-1.75) | 1.33  (0.98-1.81) | 1.39  (1.04-1.86) | 0.037 |
| Model 3 | Ref. | 1.33  (0.99-1.79) | 1.16  (0.85-1.57) | 1.28  (0.96-1.71) | 0.21 |

model 1: adjusted for age and sex; model 2: adjusted for model 1, body mass index, current smoker, alcohol use; model 3: adjusted for model 2, systolic blood pressure, fasting plasma glucose, total cholesterol, high density lipoprotein; low density lipoprotein

**Abbreviations:** CI, confidence interval
