# Supplementary material for: Association between neutrophil count and the risk of cardiovascular disease: A community-based cohort study in Taiwan
Source: PLoS One. 2025 May 7;20(5):e0322645. doi: 10.1371/journal.pone.0322645 (PMC12057848; doi:10.1371/journal.pone.0322645)
Supplement: S9 Table — (DOCX) [file pone.0322645.s009.docx]

**S9 Table. The cardiovascular disease incidence according to the quartiles of platelet**

| **Variables** | **Q1** | **Q2** | **Q3** | **Q4** |  |
| --- | --- | --- | --- | --- | --- |
| Participants | 728 | 749 | 732 | 746 |  |
| Person-years | 12005 | 13312 | 13202 | 13805 |  |
| Events | 97 | 102 | 98 | 103 |  |
| Incidence rate per 1000-person years | 8.08 | 7.66 | 7.42 | 7.46 |  |
| **Hazard ratio (95% CI)** | | | | | **p for trend** |
| Model 1 | Ref. | 0.995  (0.75-1.31) | 1.05  (0.80-1.40) | 1.22  (0.92-1.62) | 0.13 |
| Model 2 | Ref. | 1.02  (0.77-1.35) | 1.06  (0.80-1.41) | 1.21  (0.91-1.60) | 0.17 |
| Model 3 | Ref. | 0.98  (0.74-1.30) | 1.00  (0.75-1.32) | 1.06  (0.80-1.41) | 0.64 |

model 1: adjusted for age and sex; model 2: adjusted for model 1, body mass index, current smoker, alcohol use; model 3: adjusted for model 2, systolic blood pressure, fasting plasma glucose, total cholesterol, high density lipoprotein; low density lipoprotein

**Abbreviations:** CI, confidence interval
