# Supplementary material for: Association between neutrophil count and the risk of cardiovascular disease: A community-based cohort study in Taiwan
Source: PLoS One. 2025 May 7;20(5):e0322645. doi: 10.1371/journal.pone.0322645 (PMC12057848; doi:10.1371/journal.pone.0322645)
Supplement: S10 Table — (DOCX) [file pone.0322645.s010.docx]

**S10 Table. Subgroup analysis of the cardiovascular disease incidence according to the quartiles of red blood cell**

| **Variables** | | **Q1** | **Q2** | | **Q3** | | | **Q4** | | **p-value for interaction** |
| --- | --- | --- | --- | --- | --- | --- | --- | --- | --- | --- |
| Age |  | | |  | |  |  | | 0.43 | |
| 35–64 years old | | 1 | 0.76  (0.51-1.14) | | 0.85  (0.58-1.27) | | | 0.57  (0.38-0.88) | |  |
| ≥65 years old | | 1 | 1.07  (0.70-1.65) | | 1.12  (0.69-1.80) | | | 1.31  (0.79-2.18) | |  |
| Sex |  | | |  | |  |  | | 0.33 | |
| Men | | 1 | 1.03  (0.64-1.68) | | 0.96  (0.61-1.52) | | | 0.94  (0.59-1.51) | |  |
| Women | | 1 | 0.85  (0.57-1.24) | | 1.29  (0.87-1.93) | | | 0.92  (0.56-1.51) | |  |

Above odds ratio is adjusted by model 3 (age, sex, body mass index, current smoker, alcohol use, systolic blood pressure, fasting plasma glucose, total cholesterol, high density lipoprotein, low density lipoprotein)
