# Supplementary material for: Association between neutrophil count and the risk of cardiovascular disease: A community-based cohort study in Taiwan
Source: PLoS One. 2025 May 7;20(5):e0322645. doi: 10.1371/journal.pone.0322645 (PMC12057848; doi:10.1371/journal.pone.0322645)
Supplement: S11 Table — (DOCX) [file pone.0322645.s011.docx]

**S11 Table. Subgroup analysis of the cardiovascular disease incidence according to the quartiles of white blood cell**

| **Variables** | | **Q1** | **Q2** | **Q3** | **Q4** | **p-value for interaction** |
| --- | --- | --- | --- | --- | --- | --- |
| Age | | | | | | 0.44 |
| 35–64 years old | | 1 | 1.27  (0.85-1.92) | 1.25  (0.83-1.87) | 1.38  (0.92-2.05) |  |
| ≥65 years old |  | 1 | 0.94  (0.59-1.48) | 1.15  (0.71-1.84) | 1.16  (0.72-1.88) |  |
| Sex | | | | | | 0.52 |
| Men | | 1 | 1.33  (0.83-2.09) | 1.53  (0.97-2.42) | 1.87  (1.19-2.91) |  |
| Women | | 1 | 1.06  (0.70-1.59) | 0.98  (0.64-1.49) | 1.05  (0.68-1.63) |  |

Above odds ratio is adjusted by model 3 (age, sex, body mass index, current smoker, alcohol use, systolic blood pressure, fasting plasma glucose, total cholesterol, high density lipoprotein, low density lipoprotein)
