# Supplementary material for: Association between neutrophil count and the risk of cardiovascular disease: A community-based cohort study in Taiwan
Source: PLoS One. 2025 May 7;20(5):e0322645. doi: 10.1371/journal.pone.0322645 (PMC12057848; doi:10.1371/journal.pone.0322645)
Supplement: S12 Table — (DOCX) [file pone.0322645.s012.docx]

**S12 Table. Subgroup analysis of the cardiovascular disease incidence according to the quartiles of lymphocyte count**

| **Variables** | | **Q1** | **Q2** | | **Q3** | | **Q4** | | **p-value for interaction** |
| --- | --- | --- | --- | --- | --- | --- | --- | --- | --- |
| Age |  | |  |  | |  | | 0.45 | |
| 35–64 years old | | 1 | 1.56  (1.05-2.33) | | 1.18  (0.78-1.79) | | 1.26  (0.85-1.85) | |  |
| ≥65 years old | | 1 | 1.04  (0.66-1.62) | | 0.97  (0.61-1.55) | | 1.10  (0.69-1.75) | |  |
| Sex | | | | 0.53 | | | | | |
| Men | | 1 | 1.61  (1.05-2.45) | | 1.47  (0.95-2.27) | | 1.86  (1.22-2.83) | |  |
| Women | | 1 | 1.06  (0.70-1.61) | | 0.94  (0.60-1.46) | | 0.91  (0.60-1.38) | |  |

Above odds ratio is adjusted by model 3 (age, sex, body mass index, current smoker, alcohol use, systolic blood pressure, fasting plasma glucose, total cholesterol, high density lipoprotein, low density lipoprotein)
