# Supplementary material for: Association between neutrophil count and the risk of cardiovascular disease: A community-based cohort study in Taiwan
Source: PLoS One. 2025 May 7;20(5):e0322645. doi: 10.1371/journal.pone.0322645 (PMC12057848; doi:10.1371/journal.pone.0322645)
Supplement: S13 Table — (DOCX) [file pone.0322645.s013.docx]

**S13 Table. Subgroup analysis of the cardiovascular disease incidence according to the quartiles of platelet**

| **Variables** | **Q1** | **Q2** | | **Q3** | **Q4** | **p-value for interaction** |
| --- | --- | --- | --- | --- | --- | --- |
| Age | | | 0.81 | | | |
| 35–64 years old | 1 | 0.86  (0.59-1.24) | | 0.95  (0.66-1.37) | 0.89  (0.62-1.27) |  |
| ≥65 years old | 1 | 1.11  (0.73-1.71) | | 1.01  (0.63-1.62) | 0.995  (0.63-1.58) |  |
| Sex | | | 0.89 | | | |
| Men | 1 | 0.95  (0.66-1.39) | | 1.07  (0.73-1.57) | 1.18  (0.80-1.73) |  |
| Women | 1 | 1.00  (0.65-1.53) | | 0.90  (0.59-1.38) | 0.94  (0.62-1.43) |  |

Above odds ratio is adjusted by model 3 (age, sex, body mass index, current smoker, alcohol use, systolic blood pressure, fasting plasma glucose, total cholesterol, high density lipoprotein, low density lipoprotein)
