# Supplementary material for: Association between neutrophil count and the risk of cardiovascular disease: A community-based cohort study in Taiwan
Source: PLoS One. 2025 May 7;20(5):e0322645. doi: 10.1371/journal.pone.0322645 (PMC12057848; doi:10.1371/journal.pone.0322645)
Supplement: S14 Table — (DOCX) [file pone.0322645.s014.docx]

**S14 Table. Sensitivity analysis of the cardiovascular disease incidence according to the quartiles of red blood cell**

|  | **Red blood cell** | | | |  |
| --- | --- | --- | --- | --- | --- |
| **Variables** | **Q1** | **Q2** | **Q3** | **Q4** | **p-value for trend** |
| Exclude extreme data^a^ | Ref. | 1.01  (0.75-1.36) | 0.95  (0.69-1.30) | 0.995  (0.72-1.38) | 0.93 |
| Exclude extreme data^b^ | Ref. | 0.997  (0.74-1.34) | 1.10  (0.81-1.49) | 0.96  (0.69-1.33) | 0.84 |

a: Extreme data include: Hb>16.5 g/dL

b: Extreme data include: Platelet> 450x10^3^/μL or <100x10^3^/μL
