# Supplementary material for: Association between neutrophil count and the risk of cardiovascular disease: A community-based cohort study in Taiwan
Source: PLoS One. 2025 May 7;20(5):e0322645. doi: 10.1371/journal.pone.0322645 (PMC12057848; doi:10.1371/journal.pone.0322645)
Supplement: S15 Table — (DOCX) [file pone.0322645.s015.docx]

**S15 Table. Sensitivity analysis of the cardiovascular disease incidence according to the quartiles of white blood cell**

|  | **White blood cell** | | | |  |
| --- | --- | --- | --- | --- | --- |
| **Variables** | **Q1** | **Q2** | **Q3** | **Q4** | **p-value for trend** |
| Exclude extreme data^a^ | Ref. | 1.19  (0.87-1.63) | 1.15  (0.84-1.57) | 1.35  (0.99-1.85) | 0.07 |
| Exclude extreme data^b^ | Ref. | 1.18  (0.87-1.61) | 1.16  (0.86-1.57) | 1.30  (0.96-1.77) | 0.12 |

a: Extreme data include: Hb>16.5 g/dL

b: Extreme data include: Platelet> 450x10^3^/μL or <100x10^3^/μL
