# Supplementary material for: Association between neutrophil count and the risk of cardiovascular disease: A community-based cohort study in Taiwan
Source: PLoS One. 2025 May 7;20(5):e0322645. doi: 10.1371/journal.pone.0322645 (PMC12057848; doi:10.1371/journal.pone.0322645)
Supplement: S16 Table — (DOCX) [file pone.0322645.s016.docx]

**S16 Table. Sensitivity analysis of the cardiovascular disease incidence according to the quartiles of lymphocyte count**

|  | **Lymphocyte count** | | | |  |
| --- | --- | --- | --- | --- | --- |
| **Variables** | **Q1** | **Q2** | **Q3** | **Q4** | **p-value for trend** |
| Exclude extreme data^a^ | Ref. | 1.08  (0.79-1.47) | 1.08  (0.78-1.50) | 1.12  (0.82-1.54) | 0.52 |
| Exclude extreme data^b^ | Ref. | 1.06  (0.78-1.43) | 1.03  (0.75-1.42) | 1.12  (0.82-1.52) | 0.49 |

a: Extreme data include: Hb>16.5 g/dL

b: Extreme data include: Platelet> 450x10^3^/μL or <100x10^3^/μL
