# Supplementary material for: Association between neutrophil count and the risk of cardiovascular disease: A community-based cohort study in Taiwan
Source: PLoS One. 2025 May 7;20(5):e0322645. doi: 10.1371/journal.pone.0322645 (PMC12057848; doi:10.1371/journal.pone.0322645)
Supplement: S17 Table — (DOCX) [file pone.0322645.s017.docx]

**S17 Table. Sensitivity analysis of the cardiovascular disease incidence according to the quartiles of platelet**

|  | **Platelet** | | | |  |
| --- | --- | --- | --- | --- | --- |
| **Variables** | **Q1** | **Q2** | **Q3** | **Q4** | **p-value for trend** |
| Exclude extreme data^a^ | Ref. | 0.99  (0.74-1.32) | 0.98  (0.73-1.32) | 1.10  (0.82-1.48) | 0.50 |
| Exclude extreme data^b^ | Ref. | 0.97  (0.73-1.29) | 0.98  (0.74-1.31) | 1.03  (0.77-1.37) | 0.81 |

a: Extreme data include: Hb>16.5 g/dL

b: Extreme data include: Platelet> 450x10^3^/μL or <100x10^3^/μL
