# Supplementary figures and images for: Association between neutrophil count and the risk of cardiovascular disease: A community-based cohort study in Taiwan
Source: PLoS One. 2025 May 7;20(5):e0322645. doi: 10.1371/journal.pone.0322645 (PMC12057848; doi:10.1371/journal.pone.0322645)

**S1 Figure. Flow chart of study population selection.**


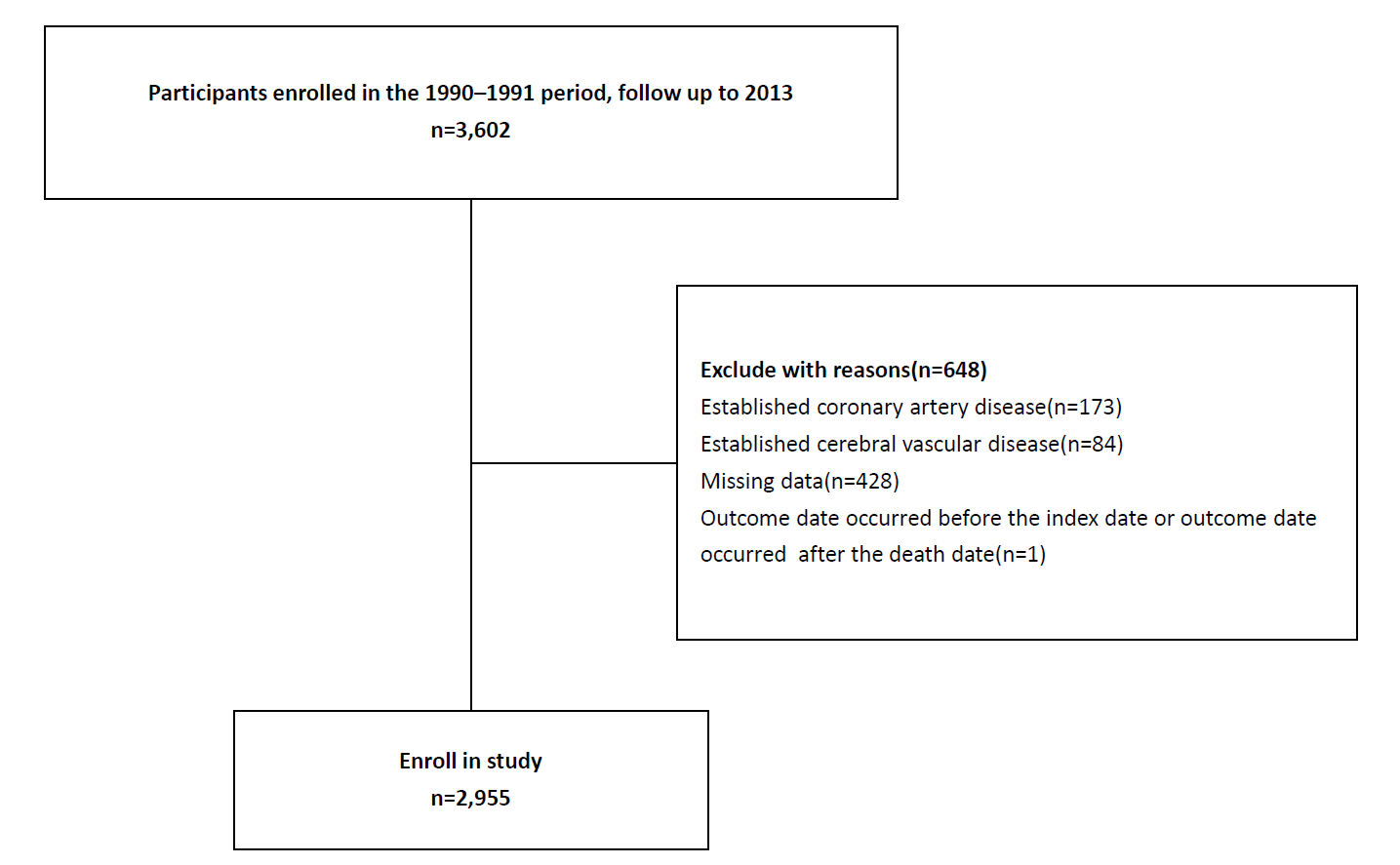

Supplement: S1 Fig — (DOCX) [file pone.0322645.s018.docx]
