# Supplementary material for: Association between neutrophil count and the risk of cardiovascular disease: A community-based cohort study in Taiwan
Source: PLoS One. 2025 May 7;20(5):e0322645. doi: 10.1371/journal.pone.0322645 (PMC12057848; doi:10.1371/journal.pone.0322645)
Supplement: S3 Fig — (DOCX) [file pone.0322645.s020.docx]

**S3 Figure. The Kaplan-Meier survival curves for cardiovascular disease of WBC**
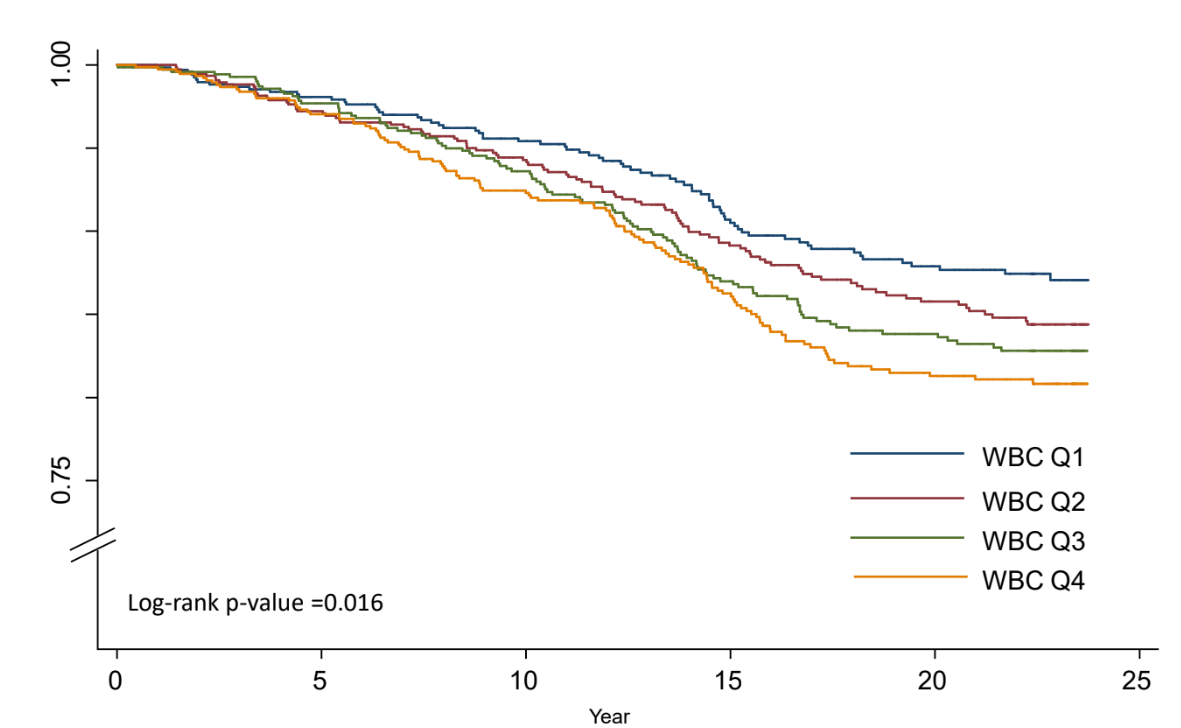


**Abbreviations:** WBC, white blood cell
