# Supplementary material for: Association between neutrophil count and the risk of cardiovascular disease: A community-based cohort study in Taiwan
Source: PLoS One. 2025 May 7;20(5):e0322645. doi: 10.1371/journal.pone.0322645 (PMC12057848; doi:10.1371/journal.pone.0322645)
Supplement: Attachment — Submitted filename: Response to Reviewers.docx [file pone.0322645.s024.docx]

**Response to Reviewer #1**

Dear Reviewer,

Thank you for your thoughtful comments and valuable suggestions, which have greatly contributed to improving the clarity and quality of our manuscript. Below, we address each of your comments in detail and outline the revisions made to the manuscript:

**1.** To strengthen the validity of your findings, consider including a sample size estimation or a post-hoc power calculation for the primary outcome. This addition will provide insight into the study's statistical power and robustness.

**Response:** Thank you for the suggestion. I have added a sample size estimation in the Methods section. We set the alpha level at 0.05 and the power (1−β) at 0.80, assuming a ratio of Neutrophil Q4 to Q1 of approximately 1:1. Based on previous literature, 10.6% incidence of CVD in Q1, and a relative risk (RR) of 1.57 for Q4 compared with Q1(ref.). Under these assumptions, our statistical software indicated that a total of 1,380 participants would be required.

Since our study included 2,955 participants—substantially higher than this estimated requirement—it is likely that we had adequate statistical power to detect the main effect of interest. This supplementary analysis therefore strengthens our primary findings and underscores the robustness of our conclusions.

Ref: Karino, S., et al., *Total and differential white blood cell counts predict eight-year incident coronary heart disease in elderly Japanese-American men: the Honolulu Heart Program.* Atherosclerosis, 2015. **238**(2): p. 153-8.

**2.** It would be beneficial to clarify whether certain factors in your analysis were treated as covariates or confounders. Reviewing the existing literature to appropriately classify these factors and using precise terminology will improve the study's methodological rigor. Although the overall analysis is correct.

**Response:** Thank you for the suggestion. I have added references for the covariates used in this study at lines 134–137 to ensure clarity and to provide appropriate citations for the variables included in the analysis.

**3.** Given the presence of multiple comparisons/testing and stepwise analyses in the study, it is crucial to adjust the standard significance level (e.g., using Bonferroni correction) to mitigate the risk of Type I errors. Such adjustments might also impact the reported confidence intervals and should be considered.

**Response:** Thank you for your suggestion. In Table 2, we did not use ANOVA for our comparisons; rather, we used p-value for trend analysis to assess whether there is a linear increase in CVD risk as neutrophil count rises. This approach avoids the multiple comparisons issue that ANOVA can sometimes pose, and thus does not require a correction for multiple testing.

**4.**On page 10 (line 203), the term "multivariate Cox regression models" is used. The appropriate term in this context appears to be "multivariable," as it more accurately reflects the inclusion of multiple independent variables rather than dependent ones.

**Response:** Thank you for the reviewer’s suggestion. I have replaced "multivariate" with "multivariable" in the manuscript to ensure the terminology is accurate and appropriate.

**5–7.**

- Including interpretation zones and cut-offs for hazard ratios (HR), supported by references, will enrich the methods and discussion sections. This approach can help contextualize the findings more effectively for the audience.
- The dose-response trend in HR values observed in Table 2 is noteworthy. Highlighting this trend in your interpretation will add depth to the results and strengthen the discussion.
- The discussion section would benefit from using simple words for readers. For instance, rather than merely stating an HR of 1.72, explain that this translates to a 72% higher hazard in group 4 compared to group 1. Such simplifications will enhance reader comprehension.

**Response:** Thank you for the reviewer’s suggestions. I have revised the first paragraph of the “Discussion” to provide a more detailed explanation of the significance of increasing HR values and to elaborate on the dose–response trend observed in Table 2. Additionally, I have simplified the interpretation of the results to improve reader comprehension, such as explaining that an HR of 1.72 corresponds to a 72% higher hazard in group 4 compared to group 1.

**Response to Reviewer #2:**

Dear Reviewer,

Thank you for your thoughtful comments and valuable suggestions, which have significantly helped improve the quality of our manuscript. Below, we address each of your comments in detail and outline the revisions made to the manuscript.

**Reviewer Comment:** It is a good article, but almost the same article was done in 2023, which is available on the Internet. Therefore, your article should investigate the topic further or the discussion and conclusion section should further investigate and explain the mechanism of connection and correlation. In my opinion, make the discussion and conclusion section more complete.
Good luck.

**Response:** Thank you for your insightful suggestion. I have reviewed the 2023 article on the topic of neutrophil counts and cardiovascular disease and incorporated its relevant findings into the discussion. While the topic and main findings are consistent with our study, our research focuses on a cohort of Chinese adults followed over an extended 20-year period. This long-term follow-up allowed us to capture the natural history of cardiovascular disease in a stable environment, providing robust observational insights that complement genetic instrument-based approaches.

**Response to Reviewer #3**

Dear Reviewer,

Thank you for your thoughtful comments and valuable suggestions, which have significantly helped improve the quality of our manuscript. Below, we address each of your comments in detail and outline the revisions made to the manuscript.

1.**Reviewer Comment:** I would like to congratulate the authors for raising the important topic pertaining to patient-centered care. When reading the introduction and discussion parts, I found them exciting and interesting. I hope this paper will contribute to new knowledge by introducing the association between neutrophil count and the risk of cardiovascular disease.

**Response:** Thank you for your kind words and positive feedback on our study. We are encouraged by your comments and have addressed the specific issues you raised below.

2.**Reviewer Comment:** Work extensively to clear grammar and typographical errors throughout the document.

**Response:** Thank you for the suggestion. I have carefully reviewed the entire document and made extensive revisions to address grammatical issues and typographical errors. These changes ensure that the manuscript is more precise and reader-friendly while maintaining the integrity of the original findings.

**3. Abstract**

**Reviewer Comment:**

- Revise the background, as I noted information is mixing and not needed in this part (Line 30-40).
- Revise the conclusion to ensure it aligns with the results.

**Response:** Thank you for the suggestion. I have revised the abstract based on the study’s conclusions to improve clarity and ensure smoother phrasing.

**4. Introduction**

**Reviewer Comment:**

- The authors should explain the scientific background and rationale for the investigation being reported.
- Line 90: What does "CCCC" mean?
- State specific objectives, including any prespecified hypotheses.

**Response:** Thank you for the suggestion. I have revised the introduction to more clearly explain the rationale for the investigation and the hypothesis. Additionally, the database used in this study, derived from the Chin-Shan Community Cardiovascular Study (CCCC), has been described in greater detail in the Methods section.

**5. Methods**

**(1) Reviewer Comment:**

- Clarify whether this is a retrospective or prospective study.
- Line 124 needs to be revised for clarity.

**Response:** Thank you for the suggestion. I have added a statement in the manuscript clarifying that this study is a retrospective study. Additionally, I have revised Line 131-132(new location) to make the sentence clearer.

**(2) Reviewer Comment:**

- Provide the eligibility criteria, and the sources and methods used for selecting participants. Describe methods of follow-up.

**Response:** Thank you for the suggestion. I have revised the Methods section to provide a clearer description of the eligibility criteria, as well as the sources and methods used for selecting participants.

**(3) Reviewer Comment:**

- For each variable of interest, give sources of data and details of methods of assessment (measurement). Describe comparability of assessment methods if there is more than one group.

**Response:** Thank you for the suggestion. I have revised the Methods section to provide a clearer description of the participants’ underlying diseases and baseline health status, including age, sex, smoking history, and alcohol consumption, which were collected using standardized questionnaires. Laboratory data, including complete blood counts and metabolic-related markers, were obtained by the same technicians at the same medical facility during the 1990–1991 baseline period.

**(4) Reviewer Comment:**

- Explain how the study size was determined.

**Response:** Thank you for the suggestion. I have added a sample size estimation in the Methods section. We set the alpha level at 0.05 and the power (1−β) at 0.80, assuming a ratio of Neutrophil Q4 to Q1 of approximately 1:1. Based on previous literature, 10.6% incidence of CVD in Q1, and a relative risk (RR) of 1.57 for Q4 compared with Q1(ref.). Under these assumptions, our statistical software indicated that a total of 1,380 participants would be required.

Since our study included 2,955 participants—substantially higher than this estimated requirement—it is likely that we had adequate statistical power to detect the main effect of interest. This supplementary analysis therefore strengthens our primary findings and underscores the robustness of our conclusions.

Ref: Karino, S., et al., *Total and differential white blood cell counts predict eight-year incident coronary heart disease in elderly Japanese-American men: the Honolulu Heart Program.* Atherosclerosis, 2015. **238**(2): p. 153-8.

**6. Results**

**Reviewer Comment:**

- Provide unadjusted and confounder-adjusted estimates and their precision (e.g., 95% confidence interval). Clearly indicate which confounders were adjusted for and why they were included.
- Report category boundaries when continuous variables were categorized.
- If relevant, consider translating estimates of relative risk into absolute risk for a meaningful time period.

**Response:** Thank you for the suggestion. I have added the unadjusted hazard ratio to Table 2. In addition, the neutrophil count category boundaries are as follows: Q1 (0.7–3.0), Q2 (3.0–3.8), Q3 (3.8–4.7), and Q4 (>4.7–41.3 × 10³/mL). These boundaries are provided in the second paragraph of the Results section and in Table 1.

Furthermore, regarding absolute risk, Table 2 already presents the incidence rates per 1,000 person-years for Q1 to Q4, which should effectively convey the overall risk to the readers.

**7. Discussion**

**Reviewer Comment:**

- Summarize key results with reference to study objectives.
- Improve the discussion of limitations.

**Response:** Thank you for the suggestion. I have revised the Discussion section to provide a clearer summary of the key results in the first paragraph. Additionally, I have refined the limitations section to make the content more comprehensive and detailed.
