# Supplementary material for: Association between neutrophil count and the risk of cardiovascular disease: A community-based cohort study in Taiwan
Source: PLoS One. 2025 May 7;20(5):e0322645. doi: 10.1371/journal.pone.0322645 (PMC12057848; doi:10.1371/journal.pone.0322645)
Supplement: Attachment — Submitted filename: Response_to_Reviewers_auresp_2.docx [file pone.0322645.s025.docx]

**Response to Reviewer #3**

Dear Reviewer,

Thank you for your helpful comments and suggestions. They have helped us improve the clarity and overall quality of our manuscript. Below, we provide our responses to each of your points and explain the revisions.

1. **Reviewer Comment:**
   Tittle: The title is quite explicit, but I observed that in line 4, there is no need to rewrite the title.

**Response:** Thank you for your suggestion. The text in line 4 is the short title, which is one of the requirements of PLOS ONE to help readers quickly understand the key points of the study's topic.

2. **Abstract**

**Reviewer Comment:**

A. Background: it is unnecessary to include the design on a portion of the background; instead, you should condense the problem statement and the objective of your research.

**Response:** Thank you for your suggestion. I have revised the Background section of the abstract to make it more concise and focused on the research problem and objective. Below is the revised paragraph:

"Neutrophil count is associated with atherosclerotic plaque formation and cardiovascular diseases (CVD). As previous studies have been predominantly conducted in Caucasians, the significance of neutrophil count as a clinical factor in CVD among other ethnic groups remains unclear."

B. Result: In the results section, the authors should begin with the findings instead of starting with demographic information.

**Response:** Thank you for your suggestion. I have revised the Results section so that it starts with the study’s main findings instead of demographic details. Below is the revised paragraph:

“Over a median follow-up period of 22 years, 400 cases of new-onset CVD were recorded. Cox proportional hazards regression analysis revealed that a higher neutrophil count was independently associated with CVD incidence in Taiwanese adults, with an HR of 1.42 (95% CI 1.03–1.94) after adjusting for multiple covariates. This association remained consistent in both the subgroup and sensitivity analyses.”

C. Conclusion: there is no need to write the design of the study the authors should conclude according to the result.

**Response:** Thank you for your suggestion. I have revised the conclusion so that it focuses on the study’s findings. Below is the revised paragraph:
“Our study demonstrated that, in the Taiwanese population, a higher neutrophil count was associated with a higher incidence of CVD over an average 22-year follow-up in individuals without preexisting CVD.”

3. **Reviewer Comment:**
   Methods: The authors need to make revisions to this section, specifically line number 129.

**Response:** Thank you for your suggestion. I have revised line 124 (new location) and checked the Methods section to improve clarity. Below is the revised paragraph:

“The endpoint of the study was the incidence of cardiovascular events ascertained from hospital records or death certificates, and participants were followed-up until the end of 2013. Cardiovascular events included those related to CAD and CVA. The definition of CAD included fatal and nonfatal CADs, confirmed through death certificate hospitalization records of conditions that required coronary artery bypass grafting (CABG) or coronary angioplasty, respectively. The definition of CVA included neurological symptoms of vascular origin lasting more than **24 hours**, resulting in hospitalization or death. CVA subtypes, including hemorrhagic and ischemic strokes, were diagnosed using imaging; however, patients with transient ischemic attacks (TIAs) were excluded. For individuals who experienced both types of events, the endpoint was defined as the time of the first event.”

4. **Reviewer Comment:**
   Result: I observed that line number 198 is unnecessary, so please eliminate it

**Response:** Thank you for your suggestion. Line 198 refers to the abbreviation for standard deviation (SD). I have removed this sentence. Please let me know if there are any other modifications needed.
